# Isolation of human explant derived cardiac stem cells from cryopreserved heart tissue

**DOI:** 10.1371/journal.pone.0176000

**Published:** 2017-04-17

**Authors:** Robyn Jackson, Seth Mount, Bin Ye, Audrey E. Mayfield, Vincent Chan, Munir Boodhwani, Ross A. Davies, Haissam Haddad, Darryl R. Davis

**Affiliations:** 1 University of Ottawa Heart Institute, Ottawa, Canada; 2 University of Saskatchewan, Saskatoon, Canada; University of Tampere, FINLAND

## Abstract

The value of preserving high quality bio specimens for fundamental research is significant as linking cellular and molecular changes to clinical and epidemiological data has fueled many recent advances in medicine. Unfortunately, storage of traditional biospecimens is limited to fixed samples or isolated genetic material. Here, we report the effect of cryopreservation of routine myocardial biopsies on explant derived cardiac stem cell (EDC) culture outcomes. We demonstrate that immediate cryopreservation or delayed cryopreservation after suspension within cardioplegia for 12 hours did not alter EDC yields, proliferative capacity, antigenic phenotype or paracrine signature. Cryopreservation had negligible effects on the ability of EDCs to adopt a cardiac lineage, stimulate new vessel growth, attract circulating angiogenic cells and repair injured myocardium. Finally, cryopreservation did not influence the ability of EDCs to undergo genetic reprogramming into inducible pluripotent stem cells. This study establishes a means of storing cardiac samples as a retrievable live cell source for cardiac repair or disease modeling.

## Introduction

Preserving high quality bio specimens for research is increasingly being performed by a number of academic, government and industrial organizations. The value of these specimens is immense. In 2012, it was estimated that the global demand for human tissue approached $700 million and this marketplace has been growing 20–30% every year since [1]. The growth of the biobank industry reflects this need. The Rand Institute estimates that 300 million samples are currently stored in the United States and this repository is growing by 20 million specimens annually [2].

Traditionally, stored biospecimens have been restricted to fixed samples or isolated DNA/RNA with live cell storage representing a minority of specimens. This finding is understandable given that, until recently, only primitive stem cell sources (such as, sperm, ova, embryos and umbilical cord blood) could be successfully stored for long periods of time prior to usage. While functional myocardial cells can be isolated from cryopreserved neonatal murine tissue [3], the extent to which this technology can be extended to adult human cardiac tissue routinely harvested during cardiac procedures is unknown.

To address this, we evaluated the influence of cryopreservation on the culture of explant derived cardiac stem cells (EDCs) from endomyocardial biopsies or atrial appendage specimens. Expanded progeny of EDCs have recently emerged as a promising cell source to treat ischemic cardiomyopathy using techniques developed to grow these cells from patient biospecimens [4]. In previous work, we have shown that ex vivo proliferated EDCs themselves possess a complementary repertoire of sub-populations capable of differentiating into cardiac lineage, secreting cardioprotective cytokines and improving post-ischemic cardiac function when delivered soon after myocardial infarction (MI) [5–8]. The value of storing autologous cardiac-derived cells is obvious as these cells capture valuable tissue-specific genetic and cellular material for disease modeling by genetic re-programming to inducible pluripotent stem cells (iPSCs) while providing a renewable platform for cardiac regeneration [5–8]. However, the upfront cost of specimen processing ($25,000/cell line) using GMP-culture conditions effectively precludes routine collection and cell line culture.

## Materials and methods

### Ethics statement

This study was conducted according to the principles expressed in the Declaration of Helsinki. The study was approved by the Human Research Ethics Board of the University of Ottawa Heart Institute. All patients provided written informed consent for the collection of samples and subsequent analysis. All animal procedures were conducted in accordance with humane animal care standards outlined in the Canada Council on Animal Care Guide to the Care and Use of Experimental Animals and were approved by the University of Ottawa Animal Care Committee.

### Cell culture

Human EDCs were cultured from atrial appendage or myocardial biopsies specimens obtained during clinically-indicated procedures after informed consent under a protocol approved by the University of Ottawa Heart Institute Research Ethics Board. All tissue samples were sectioned and randomly allocated to immediate cell culture (fresh tissue), immediate cryopreservation or delayed cryopreservation after 24 hours of refrigeration (4°C) in cardioplegia solution (lactated ringers, 2% St Thomas solution, 5 mEq NaHCO3 and 10 mEq KCl; Thermo Fisher Scientific). Tissue samples were cryopreserved to -80°C within 5% dimethyl sulfoxide, 6% fetal bovine serum within Iscove’s Modified Dulbecco’s Medium [9]. One month later, cryopreserved tissue specimen vials were recovered in a 37°C water bath prior to processing. As previously described [5–8, 10, 11], tissue was minced, digested (1mg/ml, collagenase IV) and plated within media (Iscove’s Modified Dulbecco’s Medium, 20% fetal bovine serum, 100 U/ml penicillin G, 100 ug/ml streptomycin, 2 mmol/l L-glutamine and 0.1 mmol/l 2-mercaptoethanol; Thermo Fisher Scientific). Loosely-adherent cells that emerged from the plated cardiac tissue were harvested once a week for 4 weeks using mild enzymatic digestion (0.05% trypsin; Thermo Fisher Scientific). EDCs sourced from atrial appendages were used for all experimental procedures while EDCs from ventricular biopsies were used solely for cell counts to evaluate the effect of tissue source on the ability to proliferate cells from plated tissue.

Human circulating angiogenic cells (CACs) were isolated from patient peripheral blood samples obtained after informed consent under a protocol approved by the University of Ottawa Heart Institute Research Ethics Board using standard culture techniques [6]. Briefly, mononuclear cells were isolated using density-gradient centrifugation (Histopaque 1077; Sigma-Aldrich) and placed in culture for 4–6 days in endothelial basal media (Clonetics) supplemented with EGM-2-MV-SingleQuots (Clonetics). CACs were harvested by mechanical dissociation and were used for experimentation within 7 days of culture.

Commercially sourced human umbilical vein endothelial cells (HUVECs) were cultured per the manufacturer’s directions (Lonza).

### In vitro measures of cell identity/performance

Cell counts were performed manually in triplicate using a hematocytometer. Flow cytometry (Guava easyCyte 8HT, Millipore) was used to profile the phenotypic signature of EDCs (555596, Biolegend; FAB332A, RD Systems; 555596, BD Biosciences) using appropriate isotype controls. Conditioned media was obtained after 48 hours of culture in hypoxic (1% oxygen) low serum (1% serum) conditions to simulate the environment of the infarcted myocardium. Cytokine production was evaluated using a custom multiplex immunoassay (Luminex, Affymetrix) for the 4 most abundant cytokines produced by human EDCs [6]. All immunosorbent measures were normalized to media volume and protein content obtained from cell lysates. The effects of cryopreservation on the pro-angiogenic capacity of EDC-conditioned media was evaluated by measuring the cumulative tubule length (Image J, NeuronJ plug-in; National Institutes of Health) acquired by human umbilical vein endothelial cells after plating on a growth factor depleted matrigel assay (ECM625, Millipore) within EDC conditioned media or a serum free 100 ng/ml vascular endothelial growth factor control [5, 6, 8]. The influence of cryopreserved EDC conditioned media on the recruitment of hematological cells was evaluated using random field (6 random fields/well; Image J; Center for Bio-Image) quantification of CAC migration over 24 hours through a transwell membrane after fixation (4% paraformaldehyde) and staining with 4',6-diamidino-2-phenylindole (DAPI, Sigma-Aldrich) [6, 8, 10]. The influence of cryopreservation on the ability of EDCs to adopt a cardiac lineage was evaluated after 7 days of culture in cardiogenic media (Dulbecco's Modified Eagle's Medium—low glucose, 40% MCDB-201, 0.75% dimethylsulfoxide, 0.1% 10 mmol/l L-ascorbic acid, 0.01% ITS liquid media supplement, 0.01% linoleic acid-albumin, 0.01% Pen-Strep, 0.0002% 0.25 mmol/l dexamethasone, 0.001% 2-mercaptoethanol, 10 ng/ml recombinant mouse fibroblast growth factor 8b, 100 ng/ml fibroblast growth factor 4, 10 ng/ml recombinant human protein rhDKK-1 and 10 ng/ml recombinant human bone morphogenetic protein 2; Thermo Fisher Scientific) prior to flow cytometry (α-SMA; ab7817, Abcam), cardiac troponin T (cTnT; ab10214, Abcam) and Von Willebrand factor (vWF; ab8822, Abcam) evaluation [6, 7, 10–12].

### In vivo measures of EDC regenerative performance

Male non obese diabetic severe combined immunodeficient (NOD-SCID, Charles River) mice (8 weeks old) underwent ligation of the left coronary (LC) artery under physiological temperature control in accordance with the Canadian Council on Animal Care Guidelines for the Care and Use of Experimental Animals [6, 8, 10, 11]. One hour prior to surgery, all animals received subcutaneous buprenorphine (0.05 mg/kg) and meloxicam (1 mg/kg). Immediately before to the start of surgery, all animals received a subcutaneous dose of slow release buprenorphine (1.2 mg/kg). After surgery, animals were recovered in a 30°C incubator with supplemental oxygen and moistened food. Once they had returned to normal physiological state, they were returned to their normal holding room. Mice were housed in ventilated cages (Techniplast) with at least weekly cage changes and at least twice weekly water bottle changes. All animals were fed as much and as often as desired (Envigo). Overhead lighting within the holding rooms alternated every 12 hours between light and dark cycles. All animals were monitored by a University of Ottawa Animal Care Technician trained in the care of experimental animals at least twice daily for 3 days immediately after the surgery and then at least once every day until the end of the study. For 3 days after surgery, all animals received a daily subcutaneous dose of meloxicam (1 mg/kg). For early/humane endpoints, the University of Ottawa Animal Care Committee policy regarding endpoints and intervention for laboratory animals was strictly followed. These signs included: 1) seriously impaired ambulation (unable to reach food or water easily) and/or inability to remain upright, 2) lack of responsiveness to manual stimulation, 3) rapid weight loss or net weight loss of more than 20% of the body weight, 4) prolonged inappetence, 5) evidence of muscle atrophy/marked loss of body condition, 6) Neurological signs that included lethargy, weakness, non-coordination, head tilt, unconsciousness, seizures, circling, ataxia, stereotypic behavior and paresis or paralysis that would prevent the animal from eating, drinking or standing normally, 7) respiratory signs that included difficult or abnormal respiration, coughing, nasal discharge, cyanosis, 8) jaundice and/or anemia, 9) Unexplained/uncontrollable bleeding from any site on the body, 10) Persistent vocalization, self-mutilation, 11) increased aggression, 12) any obvious prolonged illness including such signs as chronic diarrhea or constipation, markedly discolored urine, polyuria or anuria, markedly abnormal body temperature, pale mucous membranes, hunched posture, severe dehydration, erected and neglected fur, and 13) any diagnostic result that indicates a painful or distressing pathology for which treatment is impossible, impracticable or unacceptable for experimental purposes. One week after surgery, animals were randomized to echocardiographic (VisualSonics V1.3.8) guided intra-myocardial injection of 100,000 EDCs or vehicle (saline) into the infarct border and cardiac apex regions. Left ventricular ejection fraction was evaluated 21 and 28 days after LC ligation. All functional evaluations were conducted and analyzed by investigators blinded to the animal’s treatment group. After the final assessment of myocardial function, mice were sacrificed under 5% isoflurane anesthesia using cervical dislocation once the animal was in a deep surgical plain of anesthesia as indicated by absence of withdrawal reflex to toe pinch. Hearts were then fixed with 4% paraformaldehyde for sectioning. No animals died or were euthanized for humane endpoints prior to the final assessment of myocardial function. Tissue viability was assessed using Masson’s trichrome (Thermo Fisher Scientific). Three sections from equivalent distances to the LC ligature were analyzed per animal. ImageJ software was used to measure infarct thickness in Masson's trichrome-stained hearts with 3 measurements per section taken and averaged. Transplanted cell fate was evaluated using random field analysis of immunofluorescent stained sections (3 fields within 3 sections per animal) for α-SMA, cTnT, human nuclear antigen (HNA; SAB4500768, Sigma) and vWF. Capillary density within the infarct border zone was assessed by staining for isolectin B4 expression (B-1205; Vector Laboratories) in conjunction with DAPI using one random image field per slide within the border zone assessed for isolectin B4 expression (3 equidistant slides per animal). Quantitative PCR for retained human alu sequences within the left ventricle of a subset of animals was used to evaluate long-term EDC retention [6, 8, 10, 11, 13].

### Induced pluripotent reprogramming and characterization

EDCs sourced from fresh, early cryopreserved and delayed cryopreserved tissue underwent pluripotent reprogramming using retrovirus-mediated (pMXs-hOCT4, pMXs-hSOX2, pMXs-hKLF4 and pMXs-hc-MYC; Addgene) somatic gene transfer [14, 15]. To generate VSV-G pseudotyped retrovirus, GP-2 cells were transfected using poly-ethylenimine (Thermo Fisher Scientific) according to the Manufacturer’s directions prior to retrovirus collection and filtering. Polybrene (Thermo Fisher Scientific) facilitated transduction of 5X10^4^ EDCs with 500 μl of each transcription factor retrovirus was performed and cells were maintained in standard EDC growth media (outlined above). Seven days later, cells were replated onto matrigel (Corning) coated plates followed by media change to E7 media (Stem Cell Technologies). Spent medium was replaced everyday thereafter until colonies that morphologically resembled human embryonic stem cells emerged. These colonies were mechanically collected and replated onto matrigel. iPSCs were mechanically dissociated for 5 passages and then adapted to Gentle Cell Dissociation Reagent (Stem Cell Technologies) harvest for passaging 10 times prior to experimentation.

The phenotype of iPSCs sourced from fresh, early cryopreserved and delayed cryopreserved EDCs was compared to fresh EDCs or H9 cells using live cell staining (Tra-I-60 and SSEA-4; Millipore), flow cytometry (Oct4, TRA-1, SSEA4; Millipore Technologies) and quantitative polymerase chain reaction (Oct4, Nanog, Rex1 and Dnmt3b; IDT Technologies).

### Statistical analysis

All data is presented as mean ± SEM. Data was analyzed by a one-way ANOVA followed by Bonferroni’s corrected t-test (GraphPad Prism v6.07). In all cases, variances were assumed to be equal and normality was confirmed prior to further post-hoc testing. A final value of P≤0.05 was considered significant for all analyses.

## Results

### Tissue cryopreservation does not influence cell culture outcomes

Twelve patients donated atrial appendages (n = 8; 58±2 years old; 75% male) or endomyocardial biopsies (n = 4; 66±3 years old; 67% male) for the study (Table 1). Patients who donated atrial appendages during clinically indicated surgery had a history of stable cardiac disease with cardiovascular risk factors that included diabetes (25%), hypertension (75%) and dyslipidemia (75%). The majority of patients had a history of coronary artery disease (75%) and MI (75%) while only a minority had valvular heart disease requiring surgery (25%). All patients were on stable cardiac medications for at least 3 months prior to surgery. In contrast, endomyocardial ventricular biopsies were donated by patients undergoing routine post-transplant surveillance. These patients averaged 1.1±0.6 years post-transplant, with no evidence of rejection while being maintained on standard immunosuppressive treatment. Each tissue specimen was divided into 3 sections by mass for EDC culture as fresh tissue (fresh EDCs), immediately cryopreserved tissue (early cryopreserved EDCs) or tissue cryopreserved after suspension in cardioplegia for 12 hours (delayed cryopreserved EDCs). The latter strategy was included to evaluate the influence of prolonged transport from the site of tissue harvest to a remote biobank facility. Both cryopreserved tissue sources were stored for 4 weeks before starting cell culture. EDCs sourced from atrial appendages were used for all experimental procedures while EDCs from ventricular biopsies were used solely for cell counts to evaluate the effect of tissue source on the ability to proliferate cells from plated tissue.

**Table 1:** Clinical Characteristics of patient donating tissue for the study. BMI, Body Mass Index; MI, Myocardial infarction; LV, Left Ventricle; NYHA, New York Heart Association; CCS, Canadian Cardiovascular Society; ACEI, Angiotensin Converting Enzyme Inhibitor; ARB, Angiotensin II Receptor Blocker.

	Atrial appendage patients (N = 8)		Endomyocardial biopsy patients (n = 3)
Age (yrs)	58±2	Age (yrs)	66±3
BMI (kg/m^2^)	33±3	Time from transplant to biopsy (yrs)	1.1±0.6
Sex (%male)	75%	Sex (%male)	67%
Hypertension	75%	Hypertension	67%
Dyslipidemia	75%	Dyslipidemia	100%
Ongoing Smoking	33%	Ongoing Smoking	0%
Diabetes	25%	Diabetes	0%
Thyroid disease	25%	Thyroid disease	0%
History of MI	0%	Rejection on biopsy	0%
Valvular Heart Disease	25%	Creatinine (umol/L)	67±6
Coronary Artery Disease	75%	Medications:	
Congestive Heart Failure	0%	Anti-platelet therapy	100%
LV ejection fraction	54±7	Beta-Blocker	0%
NYHA class	1.9±0.3	Statins	100%
CCS class	2.3±0.7	Diuretics	0%
Creatinine (umol/L)	94±7	ACEI or ARB	33%
Medications:		Calcium channel blocker	33%
Anti-platelet therapy	100%	Antiarrhythmic drug	0%
Beta-Blocker	50%	Anticoagulant	0%
Statins	75%	Insulin	33%
Diuretics	25%	Oral hypoglycemic	33%
ACEI or ARB	50%	Tacrolimus	100%
Calcium channel blocker	75%	Mycophenolitic acid	100%
Antiarrhythmic drug	0%	Prednisone	100%
Anticoagulant	100%		
Insulin	0%		
Oral hypoglycemics	25%		

**Fig 1:**
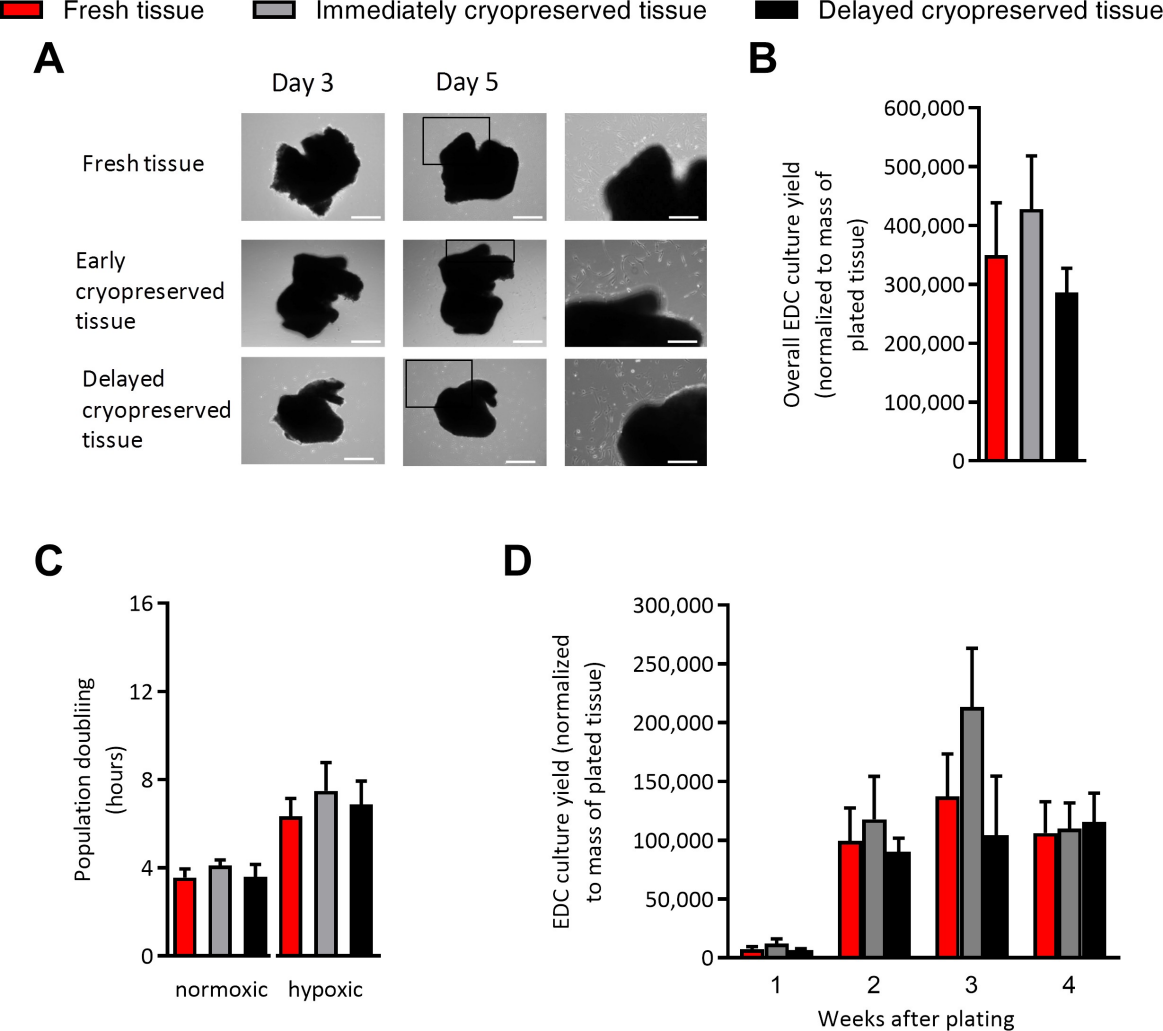
Effect of tissue cryopreservation on cell culture outcomes. (A) Representative images of plated tissue with progressive growth of EDC from days 3 to 5. Left panels provide magnified portions of the middle panels to demonstrate cells spontaneously emerging to cover the cultureware. Left and middle panels size bar is 500 μm. Right panel size bar is 250 μm. (B) Effects of tissue cryopreservation on the overall numbers of EDCs cultured from atrial appendages normalized to the mass of the plated tissue (mean ± SEM, n = 5 explant cultures). (C) Effect of tissue cryopreservation on the proliferative capacity of EDCs plated in normoxic (21% oxygen; 20% serum) and hypoxic stress (1% oxygen; 1% serum) culture conditions (mean ± SEM, n = 5 explant cultures). **(**D) Cell culture yields taken at each weekly serial harvest from plated ventricular tissue using mild trypsinization normalized to mass of the plated tissue (mean ± SEM, n = 4 explant cultures).

As shown in Fig 1A and 1B, immediate or delayed cryopreservation did not influence the time required for cells to first emerge from plated tissue or the overall cell yields from plated tissue. Similarly, when cells were collected and replated in normoxic (21% oxygen) or hypoxic (1% oxygen) conditions, immediate or delayed cryopreservation did not alter the proliferative capacity of EDCs. The ability to recover cells from cryopreservation was not restricted to atrial tissue alone as immediate and delayed cryopreservation had negligible effects on the ability of cell to emerge from plated tissue (Fig 1D).

**Fig 2:**
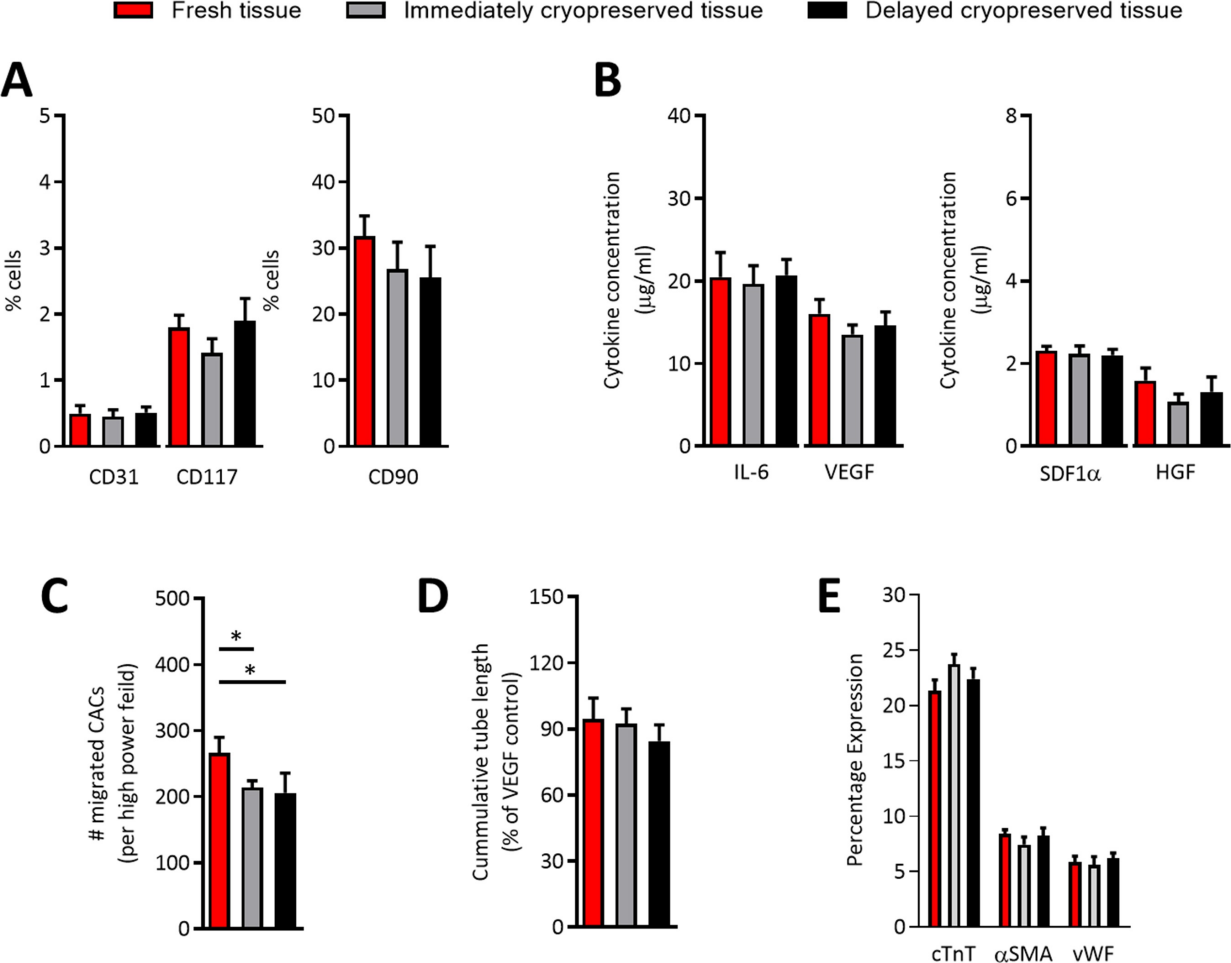
Effects of cryopreservation on the in vitro profile of EDCs. (A) Effects of tissue cryopreservation on the phenotype EDCs cultured from atrial appendages (mean ± SEM, n = 5 explant cultures). (B) Effects of tissue cryopreservation on the cytokine content within EDC conditioned media exposed to hypoxic (1% oxygen) low serum (1% serum) culture conditions (mean ± SEM, IL-6 = interleukin-6, HGF = hepatic growth factor, SDF1α = stromal cell derived factor 1α, VEGF = vascular endothelial growth factor, n = 5 conditioned media samples with 3 technical replicates). (C) Effects of tissue cryopreservation on the ability of EDC conditioned media to recruit circulating angiogenic cells (CACs) through transwell membrane (mean ± SEM, *p≤0.05, n = 5 conditioned media samples with 6 random fields/well). (D) Effects of tissue cryopreservation on the ability of EDC conditioned media to stimulate human umbilical vein endothelial cells tubule formation (mean ± SEM, n = 5 conditioned media samples). (D) Effect of tissue cryopreservation on the ability of EDCs to adopt a cardiomyocyte (cardiac troponin T, cTnT), endothelial (von Willebrand Factor, vWF) or smooth muscle (alpha smooth muscle actin, αSMA) lineage (mean ± SEM, n = 5 conditioned media samples).

Flow cytometry of representative collections from fresh and cryopreserved EDCs demonstrated that cryopreservation did not alter the phenotypic signature of EDCs (Fig 2A). Given that the majority of cell-mediated repair by cardiac-derived cells is attributable to the indirect (i.e., paracrine) mediated repair, conditioned media was collected after EDCs were plated for 48 hours in hypoxic (1% oxygen) low serum conditions (1% serum) designed to mimic ischemic myocardium. As shown in Fig 2B, secretion of the 4 most abundant cytokines produced by EDCs (i.e., was not altered by immediate or delayed cryopreservation. Similarly, application of conditioned media to in vitro assays designed to replicate angiogenesis (i.e., HUVEC tubule formation within cytokine depleted matrigel) or recruitment of circulating progenitor cells (i.e., transwell migration of CACs) demonstrated that cryopreservation did not limit EDC mediated vessel formation (Fig 2C) but had minor effects on the ability of EDCs to recruit circulating stem cells (1.3±0.1 and 1.5±0.2 fold fewer CACs recruited vs. fresh EDCs, respectively; p≤0.02; Fig 2D). Finally, the impact of cryopreservation on the cardiogenic capacity of human EDC was compared after exposure to conditions known to favor adoption of a cardiac phenotype [6, 12]. As shown in Fig 1E, cryopreservation did not alter antigenic expression of cardiac (cTnI), endothelial (vWF) or smooth muscle (αSMA) markers after exposure to inductive media known to influence the expression of cardiac markers.

### Tissue cryopreservation does not reduce EDC-mediated cardiac repair

**Fig 3:**
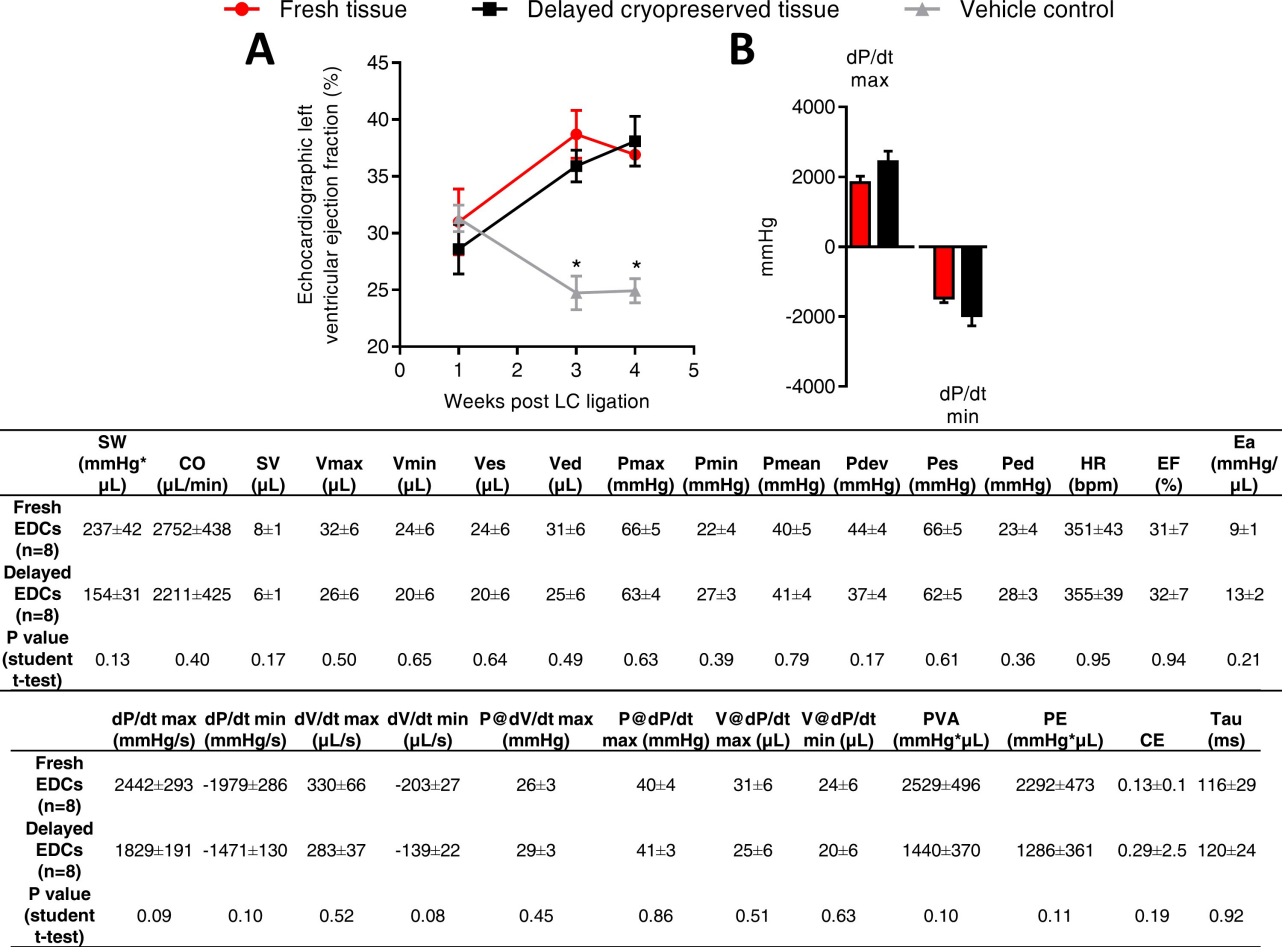
Effect of cryopreservation on cell-mediated repair of ischemic myocardium. (A) Effect of transplanting cryopreserved tissue sourced EDCs on echocardiographic function (mean ± SEM, *p≤0.05 vs. fresh or cryopreserved EDCs, n = 10–11 animals per group). (B) Effect of cryopreserved tissue sourced EDCs on invasive hemodynamics (mean ± SEM, n = 10–11 animals per group). SW, stroke work; CO, cardiac output; SV, stroke volume; Vmax, maximum volume; Vmin, minimum volume; Ves, end-systolic volume; Ved, end-diastolic volume; Pmax, maximum pressure; Pmin, minimum pressure; Pmean, mean pressure; Pdev, developed pressure; Pes, end-systolic pressure; Ped, end-diastolic pressure, HR, heart rate; EF, ejection fraction; Ea, arterial elastance; PowMax, maximum power; dP/dt max, maximum value of dP/dt; dP/dt min, minimum value of dP/dt; dV/dt max, maximum value of dV/dt; dV/dt min, minimum value of dV/dt min; P@dV/dt max, Pressure at maximum of dV/dt; P@dP/dt max, pressure at maximum of dP/dt max; V@dP/dt max, volume at maximum of dP/dt; V@dP/dt min, volume at minimum of dP/dt; PVA, pressure volume area; PE, potential energy; CE, cardiac efficiency; Tau, relaxation time constant calculated by Glantz method.

**Fig 4:**
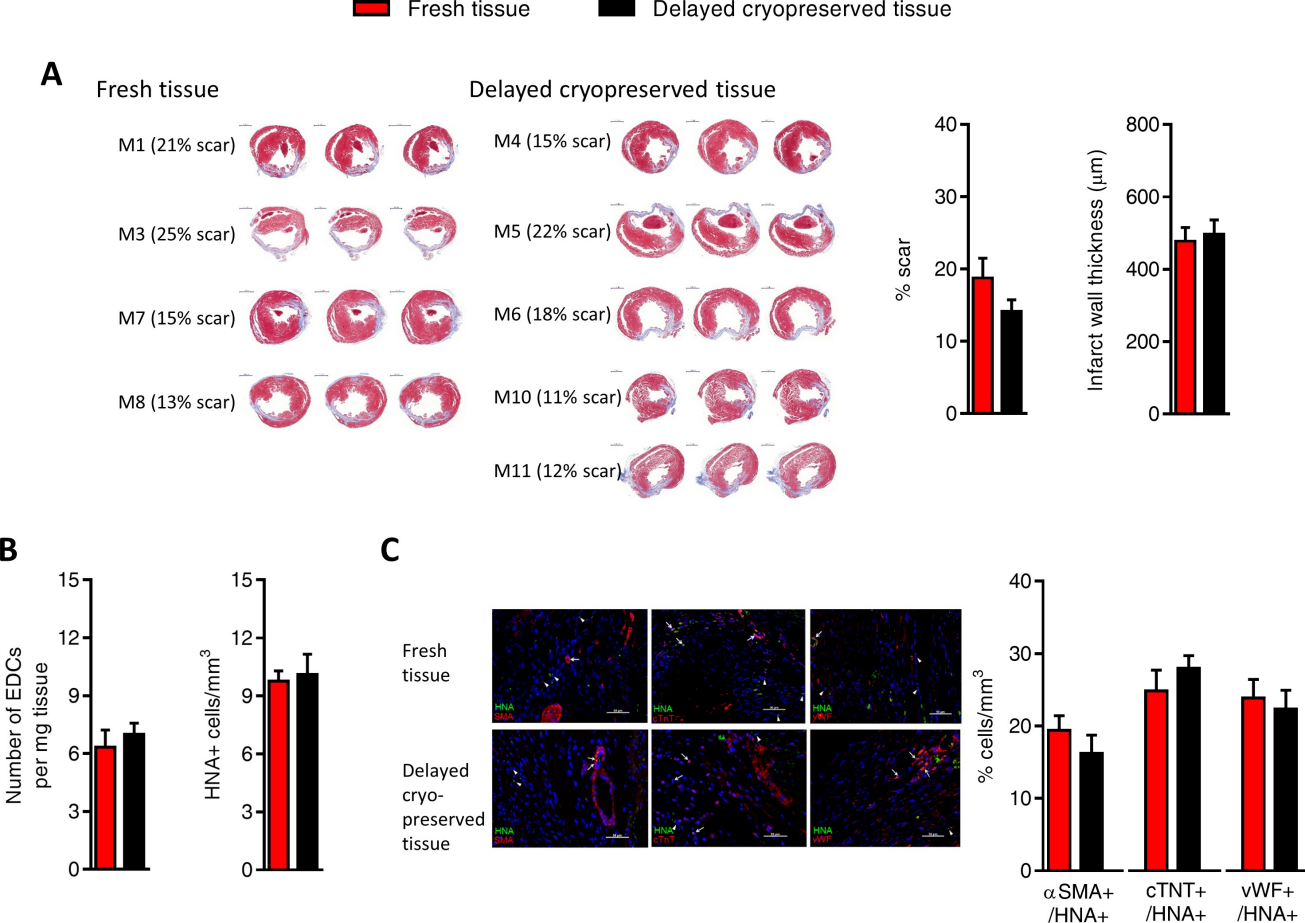
Effects of tissue cryopreservation on scar size, transplanted cell retention and transplanted cell fate. (A) Representative images (left panel) and quantitative analysis (right panels) demonstrating that tissue cryopreservation had no discernable effect on the ability of transplanted EDCs to reduce left ventricular scar sizes (mean ± SEM, n = 4–5 animals per group with 3 histological slides analyzed per animal, scale bar 1000 μm). (B) Quantitative PCR for retained human alu sequences and histological analysis for human nuclear antigen positive (HNA+) cells demonstrating that cryopreservation had no effect on transplanted EDCs engraftment (mean ± SEM, n = 5–6 animals per group). (C) Representative images of immunohistochemical sections used for peri-infarct field quantification of cell engraftment and co-localization with markers of cardiac myocyte (cTNT, upper panel), smooth muscle (αSMA, middle panel) and endothelial (vWF, lower panel) lineage (scale bar 50 μm). Arrows indicate examples of HNA co-segregation with lineage markers while arrow heads indicate HNA staining alone. Histological analysis demonstrating the co-segregation between HNA+ cells and markers of transplanted cell lineage commitment (mean ± SEM, n = 6 animals per group).

**Fig 5:**
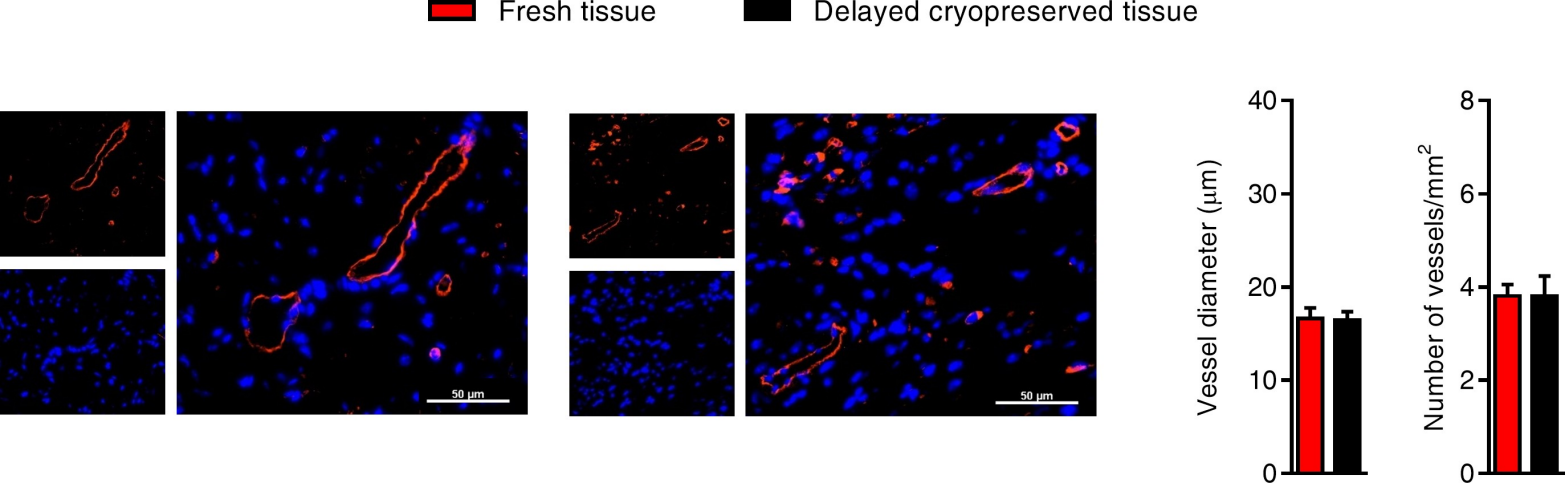
Effects of tissue cryopreservation on post-infarct neo-vascularization. Representative images (left panel) and quantitative analysis (right panels) demonstrating that tissue cryopreservation had no effect on the ability of transplanted EDCs to promote new vessel growth (mean ± SEM, n = 4–5 animals per group with 3 histological slides analyzed per animal, scale bar 50 μm).

The effect of delayed cryopreservation upon the regenerative performance of EDCs was evaluated using an immunodeficient mouse model of myocardial ischemia [5–8]. As shown in Fig 3A, animals treated with fresh or cryogenically frozen EDCs had similar echocardiographic ejection fractions 3 weeks after cell treatment which was superior to animals treated with vehicle alone. Invasive hemodynamic profiling done at the time of sacrifice mirrored this functional evaluation as there was no discernable differences between animals treated with EDCs sourced from fresh tissue or delayed cryopreserved tissue (Fig 3B). The functional benefits conferred by EDC transplantation were reflected by comparable measures of scar size and infarct wall thickness in animals treated with EDCs from fresh or delayed cryopreserved tissue (Fig 4A). Finally, cryopreservation had similar negligible effects on long-term engraftment of transplanted EDCs (Fig 4B), differentiation of engrafted EDCs (Fig 4C) and infarct neovascularization (Fig 5). Taken together, this data demonstrates that cryopreservation has negligible effects on EDC-mediated repair of ischemic myocardium.

### Tissue cryopreservation did not influence the efficiency of pluripotent reprogramming

**Fig 6:**
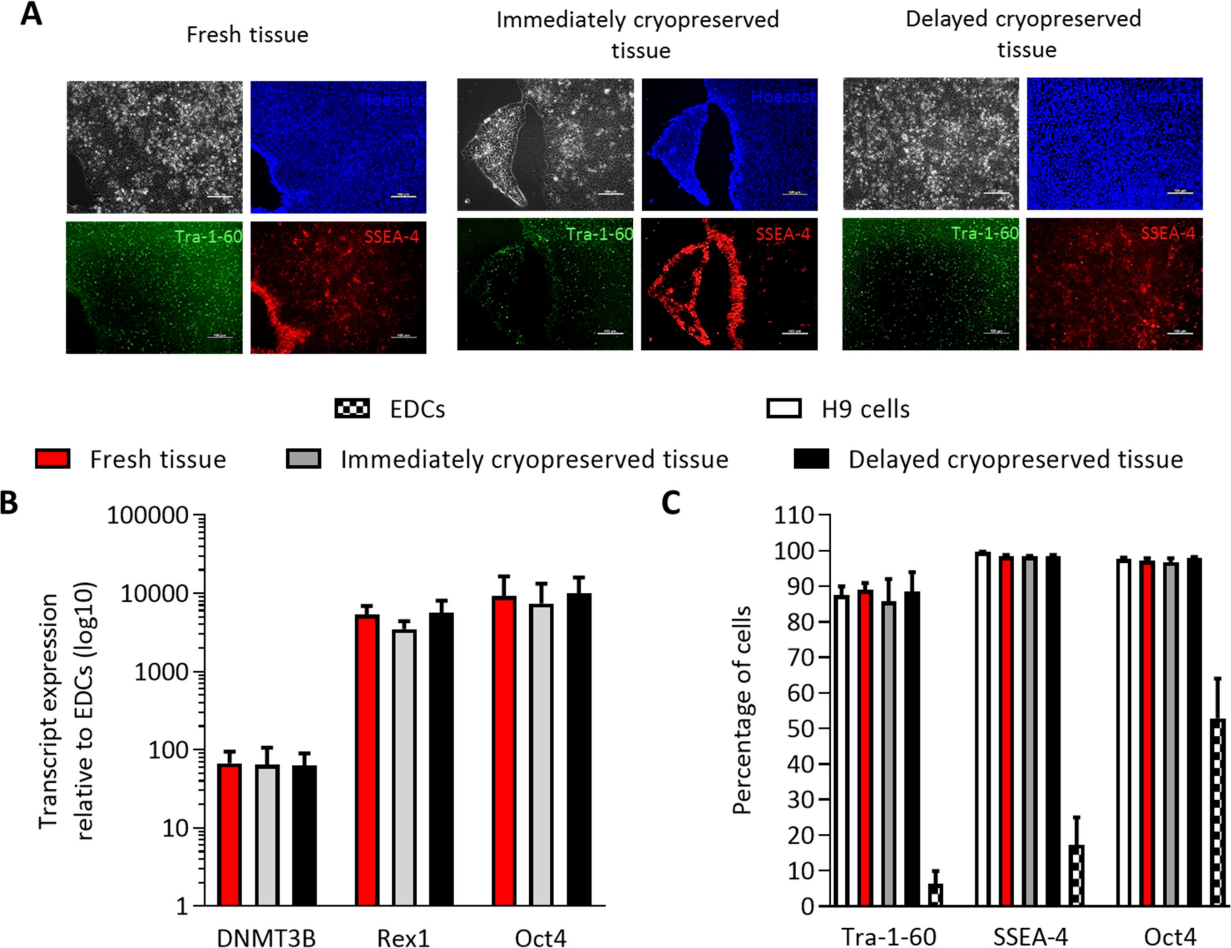
Effect of tissue cryopreservation on induced pluripotent stem cell reprogramming of EDCs. (A) Representative images of live iPSC colonies that express markers of pluripotent identity (SSEA-4 and Tra-1-60). Size bar is 100 μm. (B) Effect of tissue cryopreservation on pluripotent transcript expression of iPSC reprogrammed EDCs (mean ± SEM, n = 3 cell lines). (C) Effect of tissue cryopreservation on flow cytometry expression of pluripotent surface antigens (mean ± SEM, n = 3 cell lines).

In addition to providing an “on demand” source of autologous regenerative cells, the ability to cryopreserve clinical cardiac samples within biobanks opens the possibility of linking physiological cell models to clinical and epidemiological data. Although EDCs and sub-culutred progeny can be induced to express cardiac lineage markers[6, 10, 11, 16, 17] and electrophysiologic currents[18] characteristic of cardiomyocytes, this is infrequent and robust protocols capable of providing large numbers of contractile myocytes have not been developed. For this reason, we explored the ability of cryopreserved and fresh EDCs to undergo genetic re-programming to an iPSC phenotype. While cryopreservation of pluripotent cell lines reduces plating efficiency and clonal propagation [19–21], cryopreservation of tissue itself prior to genetic reprogramming did not influence the time whereupon the first iPSC colonies appeared or the ability to generate iPSCs from EDCs. Twenty days after reprogramming, colonies were picked based on morphological similarities to embryonic stem cells with confirmatory SSEA-4 and Tra-1-60 live-staining (Fig 6A). The expression of key pluripotency transgenes (Oct4, Rex1 and Dnmt3b) within EDCs and reprogrammed EDC-iPSCs was not influenced by cryopreservation (Fig 6B) while cryopreservation did not alter the expression of human ESC-specific surface antigens Oct4, SSEA-4 and Tra-1-60 characteristic of a pluripotent phenotype and indicating successful genetic reprogramming (Fig 6C).

## Discussion

Here, we demonstrate that cryopreservation does not alter the ability of recovered tissue biopsies to generate highly proliferative and phenotypically indistinguishable EDC lines. Cryopreservation did not influence the ability of EDC to promote angiogenesis, recruit circulating endogenous stem cells, undergo cardiogenic differentiation or provide post ischemic cell-mediated cardiac repair. Finally, cryopreservation did not alter the ability of EDCs to undergo genetic reprogramming to a primitive iPSCs lineage. Ultimately, this study goes beyond current fixed tissue storage to provide a simple, low cost means of storing live cells from the 1M+ percutaneous interventions or 400k+ cardiac surgeries done every year in North America.

The tissue sources used in this study are readily available from patients undergoing cardiac surgery or percutaneous procedures. In the 1980’s and 90’s, atrial appendage harvest was routinely performed in cardiac patients. Currently, most institutes incise the atria for bypass cannula insertion and, at the end of the case, close the incision using a purse string suture. Excising a small piece of atrial tissue, as performed in this study, has minimal risks while providing a ready tissue source for EDC culture. In contrast, ventricular cardiac biopsies were obtained from immunosuppressed patients undergoing routine post-transplant organ rejection surveillance. Extension to patients already undergoing percutaneous interventions is feasible as endomyocardial biopsy is associated with infrequent complications (<1%) that historically reflect internal jugular vein access- an adverse event which has been minimized in the era of routine ultrasound guided cannulation [22].

Finally, this study is the first to demonstrate genetic reprogramming of human EDCs to a pluripotent cell type. Although using blood, hair or skin as cell sources for reprogramming to an iPSC phenotype are considerably more convenient, human EDCs express molecular markers of early mesenchymal lineage (i.e., GATA-4, MEF2C and NKx2.5) and readily undergo genetic engineering [7, 10, 11]- a potential disposition towards attaining a pluripotent phenotype. Furthermore, these cardiac sourced cells may retain epigenetic memory or somatic-restricted mutations to provide the ideal cell source for cardiac disease modeling; however, this remains to be fully explored in future studies.

Taken together, this work provides the foundation for transitioning EDC technology towards routine use in biobank facilities as a means of providing patients and researchers with a new patient-specific tool for cardiac regeneration and disease phenotyping.
